# Underweight associated with water, sanitation, and hygiene among women of reproductive age in Arba Minch Health and Demographic Surveillance Site, Southern Ethiopia

**DOI:** 10.1002/fsn3.4184

**Published:** 2024-05-27

**Authors:** Melesse Mengesha Merkina, Tamirat Gezahegn Guyo, Desta Haftu Hayelom, Darik Temesgen Assefa, Befikadu Tariku Gutema

**Affiliations:** ^1^ Department of Health International Rescue Committee Ethiopia Program Dasenech Ethiopia; ^2^ Department of Public Health Arba Minch College of Health Sciences Arba Minch Ethiopia; ^3^ School of Public Health, College of Medicine and Health Sciences Arba Minch University Arba Minch Ethiopia

**Keywords:** body mass index, hygiene, latrine, sanitation, underweight, water

## Abstract

Ethiopia is one of the sub‐Saharan African countries where underweight among women of reproductive age is high, and it is predisposing to low birth weight, preterm birth, and reduced resistance to infections. Poor water, sanitation, and hygiene (WASH) and drinking water polluted with disease‐causing microorganisms lead to undernutrition. Therefore, this study aimed to assess the association between WASH and underweight among women of reproductive age at Arba Minch Health and Demographic Surveillance Site (HDSS), Southern Ethiopia. A community‐based cross‐sectional study was conducted at Arba Minch HDSS, South Ethiopia, from April to May 2022. Women of reproductive age were included in the study. Data were collected using a pre‐tested questionnaire and checklist. Multivariate analysis was conducted to assess the association separately for WASH indicators with underweight status of the women by adjusting for sociodemographic characteristics. Adjusted Odds Ratio (AOR) with a 95% confidence interval was used to assess the association. The prevalence of underweight and overweight/obesity among women of reproductive age was 7.82% (95% CI: 5.60, 10.81) and 12.32% (95% CI: 9.50, 15.83), respectively. Latrine hygiene and use were significantly associated with being underweight. The odds of being underweight among those who use clean latrine were 0.43 (95% CI: 0.20, 0.92) compared to those who use unclean latrine or do not have toilet facilities. The utilization of clean latrine facilities is negatively associated with being underweight among women of reproductive age. From WASH components, latrine utilization and keeping the latrine clean to use need to get focused on reducing the risk of being underweight among women of reproductive age.

## Graphical abstract

The prevalence of underweight, overweight, and obesity was 7.82% (95% CI: 5.60, 10.81) and 12.32% (95% CI: 9.50, 15.83), respectively. The odds of being underweight among those who use clean latrine were reduced by 57% (AOR = 0.43; 95% CI: 0.20, 0.92) compared to those who use unclean latrine or do not have toilet facilities. Latrine utilization and keeping the latrine clean to use need to be focused on reducing the risk of being underweight among women of reproductive age.

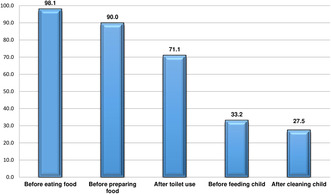

## 1 INTRODUCTION

According to the 2020 Global Nutrition Report, underweight affects nearly 462 million adults worldwide (Global Nutrition Report, 2020). The prevalence of underweight among women of reproductive age in resource‐constrained settings is increased by about tenfold when compared to those from developed countries (Abarca‐Gómez et al., 2017; Abdullah, 2015; Fanzo et al., 2019). In Sub‐Saharan Africa, where there is prolonged food insecurity, nearly 16.6% of childbearing‐age women had a low body mass index (Lartey, 2008; Razak et al., 2013). Ethiopia is one of the sub‐Saharan African countries where underweight among women of reproductive age is higher, at 22%, according to the 2016 Ethiopian demographic and health survey (EDHS) (Csace, 2016). The high prevalence of underweight among women of reproductive age groups has been related to different health problems, including low birth weight, preterm birth, stunting, and reduced resistance to infections among their offspring (Csace, 2016).

The use of contaminated or insufficient food, deprived care practices, and associated diseases are the major underlying factors that contribute to underweight. According to a survey conducted by the World Health Organization (WHO), household and environmental conditions, particularly inadequate water, sanitation, and hygiene (WASH) practices, are responsible for an estimated 50% of the global burden of malnutrition (Pruss‐Ustun et al., 2006). Poor WASH conditions increase the risk of eating food and drinking water polluted with disease‐causing microorganisms, which leads to diarrhea, parasitic infection, intestinal helminth infection, and environmental enteric dysfunction, all of which can have an impact on a person's nutritional health (Dangour et al., 2013; Fagbamigbe et al., 2017; Lin et al., 2013; Nasr et al., 2013). As a result, incorporating WASH and nutrition practices into national health policies and the strategies of development partners is critical in order to reduce the contribution of poor WASH practices to undernutrition (SUN, 2020). Previously conducted studies showed that unimproved household and environmental conditions, an unimproved source of drinking water, and an unimproved sanitary condition were significant predictors of underweight among women of reproductive age (Cairncross et al., 2010; Ferede et al., 2017; Morakinyo et al., 2020). The aim of the study was to assess the association of WASH with underweight women of reproductive age in Arba Minch Health and Demographic Surveillance Site (HDSS), Southern Ethiopia.

## 2 MATERIALS AND METHODS

### 2.1 Study area

The study was conducted in Arba Minch HDSS, Southern Ethiopia. It is located in the Arba Minch Zuria and Gaucho Baba districts of Gamo Zone. Arba Minch town, the administrative town of the Gamo Zone, is located 505 km from Addis Ababa, the capital city. The surveillance site includes nine kebeles (the lowest administrative unit of Ethiopia) of the two districts. According to a report from the site, there were 21,665 reproductive‐age women on the site in 2021.

### 2.2 Study design and population

A community‐based, cross‐sectional study was conducted from April to May 2022. Women of reproductive age residing in Arba Minch HDSS kebeles were the source population. The inclusion criteria include age from 15 to 49 years and residence in the village for at least 6 months. Pregnant and postpartum women younger than 6 months were excluded from the study.

### 2.3 Sample size determination and sampling technique

The sample size was determined by using the single population proportion formula, with a 95% confidence level, 48.6% prevalence of underweight among women of reproductive age based on the study conducted in Ziway Dugda district, Ethiopia (Ferede et al., 2017), and with 5% margin of error. By considering the assumptions mentioned above, the estimated sample size was 384. In addition, with the consideration of a ten percent nonresponse rate, the final calculated sample size was 422 women of reproductive age.

The sample size was allocated proportionally to kebeles based on the number of women of reproductive age. The sampling frame was obtained from the Arba Minch HDSS database, which contains lists of all kebele residents with their name, age, sex, and individual and household identifications. Using Stata version 14, women of reproductive age were selected randomly for each kebeles from the database of Arba Minch HDSS.

### 2.4 Data collection tools and procedures

Data were collected by using an interviewer‐administered structured questionnaire, checklists, and measurements. A checklist, which was developed by Family Health International (FHI360, 2008), was used to exclude pregnancy before starting the data collection. The questionnaire included questions about the socio‐demographic and economic characteristics of women and their households, as well as water, sanitation, and hygiene‐related characteristics. Sanitation and hygiene‐related variables include place of defecation, availability of latrine, availability of handwashing facilities, usage of latrine, handwashing practice, and the use of soap/ashes for hand washing. Water‐related questions include the availability of water, its sources, and the use of treatments. An observation checklist was used to collect information on the availability or absence of water in the home and the availability and sanitation of the latrine. Anthropometric measurements (weight and height) were used to measure the nutritional status of the women. Weight was measured using a digital floor scale (Model 869, Seca) with no shoes, and height was measured using a locally made stadiometer with participants wearing no shoes and headwear. The data collection tool was first prepared in English, translated into Amharic, and back‐translated to English to check for its consistency before administration. Nine data collectors, who have been working as data collectors at the surveillance site, collected the data. The Online Data Collection Kit (ODK) application was used to collect data.

### 2.5 Data quality assurance

Training was provided prior to data collection on the instruments, ethical issues, and the objective of the study. The height and weight of the women were measured twice, and the average of the measurements was taken. The proper operation of digital weight scales was checked daily before the start of weight measurement, and a portable standard weight of 1 kg was used to ensure the scale was exactly at zero. Prior to the data collection, a pre‐test was conducted on 5% (*n* = 21) of the sample size among individuals from one of the kebeles who were not selected as study participants. All necessary corrections were made based on the pretest results to avoid any confusion and for better completion of the questions. The data collection process was supervised by the investigator.

### 2.6 Data analysis and processing

The collected data were downloaded from the ODK aggregate as a CSV file and exported to Stata 14 for analysis. The underweight of women of reproductive age was determined based on body mass index (BMI), which was calculated as weight (kg) divided by height squared (m^2^). Those women with a BMI below 18.5 kg/m^2^ were considered underweight (WHO, 1995). An improved water source was determined based on the source of water and the treatment used. Households with a piped or protected water source (well or spring) and treated at home were considered as they use improved water sources. Otherwise, it was considered as an unimproved water source. The quality of latrine construction was determined based on the type of latrine and its construction. A household with good quality latrine construction was when the latrine type was water flash, with a ventilated improved pit latrine or pit latrine, and a house constructed with a door or lid. Poor quality latrines were those without facilities, pit latrines with houses constructed without doors and lids, and latrines without houses and slabs only. Descriptive statistics, including proportions and mean with standard deviation (SD), were performed. The household wealth index was created using principal component analysis (PCA) based on household characteristics and ownership of assets (Rutstein, 2015; Vyas & Kumaranayake, 2006). Households were classified into each of the three quantiles—poor, middle, and rich. We did bivariate and multivariate logistic regression by considering the WASH component as independent and underweight as a dependent variable. Each WASH variable was entered into multivariate logistic regression separately with potential confounding variables (age, occupation, educational status, sex of the head, and wealth index of the household). Model specification errors were checked using a link test (to test whether the regression is properly specified). The goodness of fit of the model was checked by the Hosmer–Lemeshow test. Multicollinearity was checked by using the variance inflation factor. A *p*‐value less than .05 was considered a significant relationship between the underweight and WASH variables.

### 2.7 Ethical approval

All of the study procedures were conducted in accordance with the ethical guidelines outlined in the Helsinki Declaration. Before the start of data collection, ethical approval was obtained from the Institutional Research Ethics Review Board of Arba Minch University (letter of reference: IRB/1241/2022). The School of Public Health of Arba Minch University wrote a support letter to the Gamo Zone Health Department, Arba Minch Zuria and Gacho Baba districts, and Kebele administration. A brief explanation was given about the purpose of the study, the study procedure, possible risks and benefits, and the participants' rights. Then informed consent was obtained from each study participant before asking for any information from them. For minors (age less than 18 years), written informed consent has been obtained from parents/legal guardians, and assent has been obtained from participants. No one of the study participants was obligated to participate in the study without his or her consent. They were informed that they have a full right to discontinue the study at any time if they feel uncomfortable with it. All the collected data were kept confidential, and the names or any personal identifiers of the study participants were not included in the data collection. The data were collected under COVID‐19 prevention measures.

## 3 RESULTS

### 3.1 Socio‐demographic characteristics of the respondents

A total of 422 women of reproductive age were involved in this study. The mean ± SD age of the respondents was 27.1 ± 9.09 years. Most study participants practiced protestant religion (78%) and belonged to the Gamo ethnic group (80%). Half of the respondents were housewives (47%), and around 57% were married. The average family size was 5.8 (2.0 SD) (Table 1).

**TABLE 1:** Sociodemographic characteristics of women of reproductive age in Arba Minch HDSS (*n* = 422).

Variables	Category	Freq.	%
Age (in years)	15–24	185	43.8
	25–34	138	32.7
	35–49	99	23.5
Religion	Protestant	329	78.0
	Orthodox	90	21.3
	Others^a^	3	0.7
Ethnicity	Gamo	338	80.1
	Zeyse	46	10.9
	Wolayta	27	6.4
	Others^b^	11	2.6
Occupational status	Housewife	198	46.9
	Student	128	30.3
	Merchant	20	4.7
	Others^c^	76	18.0
Marital Status	Single	164	38.9
	Married	238	56.4
	Others^d^	20	4.7
Educational status	Illiterate	144	34.1
	Primary	197	46.7
	Secondary	81	19.2
Head of the household	Male	316	74.9
	Female	106	25.1
Family size (in number)	≤5	185	43.8
	6–8	192	45.5
	9+	45	10.7

^a^ Muslim, Catholic.

^b^ Konso, Oromo.

^c^ Farmer, government employ, gospel preacher.

^d^ Divorced, Widowed.

### 3.2 Water, sanitation, and hygiene characteristics of the respondents

**FIGURE 1:**
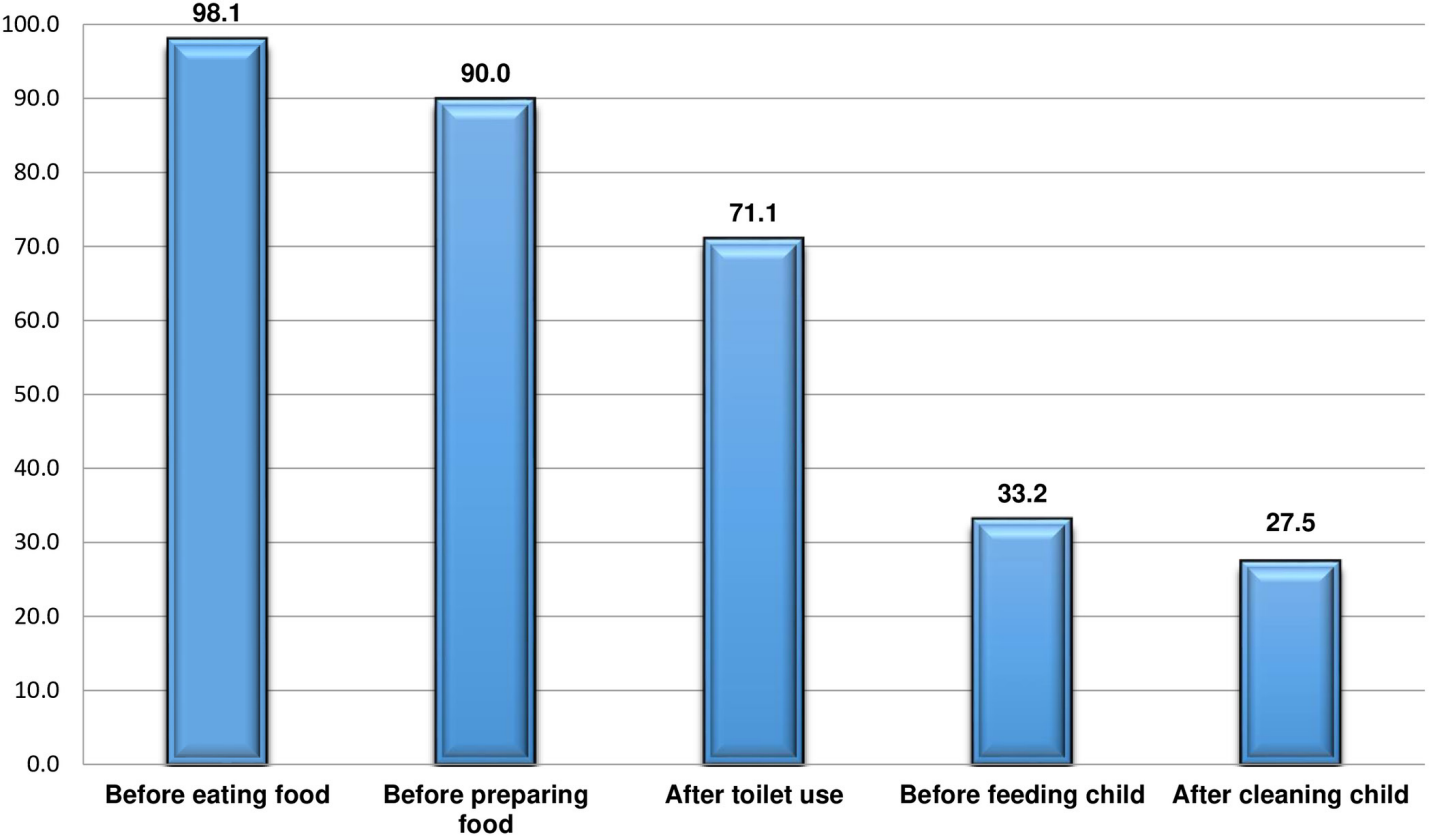
Usual wash‐your‐hand practice of the women of reproductive age at Arba Minch HDSS.

Three hundred eighteen (75.36%) households use piped water. Only 6.4% (27) of the households had a source of water within their premises. One in four (106) women of reproductive age mentioned that they treat their drinking water at home. During the visit, 75 (17.77%) households did not have drinking water in their homes. From the total participants, 19.4% (82) use shared latrine. Pit latrine was the most common (93.36%) latrine type, and 11 (2.61%) households did not have latrine facilities at all (Table 2). From the pit latrine, 10.66% (42) of the household had a ventilated, improved pit latrine, while 81.22% (320) had a house constructed and a slab on it. Twenty‐three (5.84%) and nine (2.28%) were pit with slab and only pit (no slab) latrine, respectively. One‐fifth (18.53%) of the households with pit latrines use lids to close the opening. None of the latrines were within the house. The majority (81.04%) of the latrines were located inside the compound. Out of 408 households with latrine (pit, water flush, and latrine attached to biogas), 64.93% (274) were clean and under use, while 35.07% (148) were not clean during the visit time. The remaining 15 were not in use during the visit. Overall, 80.33% of the women of reproductive age use latrines on a regular basis, while the remaining use open defecation commonly. Eighty‐three percent (350) of the households did not have a handwashing facility in the compound (Table 2). For those who had handwashing facilities in the compound, 34 had water with soap, 5 had water with ashes and 28 had water only, and 5 handwashing facility were empty during visit. Before eating was the most frequently (98.1%) mentioned time of hand washing, followed by before preparing food (90.0%) and after toilet use (71.1%) (Figure 1). Around two‐thirds (65.4%) use soap, and 34.60% (146) use only water for washing hands most of the time.

**TABLE 2:** The water, sanitation, and hygiene (WASH) characteristics of women of reproductive age in Arba Minch HDSS (*n* = 422).

Variables	Category	Freq.	%
Source of drinking water	Pipe	318	75.36
	Spring/well	98	23.22
	River	6	1.42
Where did you get drinking water for your household?	Within the premises	27	6.4
	Out of the premises	395	93.6
Is drinking water from the main source currently available?	No	75	17.77
	Yes	347	82.23
Treatment of drinking water	Yes	106	25.1
	No	316	74.9
Latrine type	Pit latrine	394	93.36
	Flush latrine	12	2.84
	Bucket latrine	3	0.71
	Attached to biogas	2	0.47
	No latrine	11	2.61
Sharing latrine	Yes	82	19.4
	No	340	80.6
Usual place to defecate for household members	Open field	83	19.67
	Pit latrine	339	80.33
Handwashing facility	No	350	82.94
	Yes	72	17.06
Usually use to wash your hand	Water only	146	34.60
	With soap	276	65.40

### 3.3 Nutritional status of the women of reproductive women

The mean (SD) BMI of the women of reproductive age was 22.04 (2.77) kg/m^2^. The study revealed that the prevalence of underweight was 7.82% (95% CI: 5.60, 10.81). The prevalence of overweight/obesity among women of reproductive age was 12.32% (95% CI: 9.50, 15.83).

### 3.4 Association between underweight and WASH in women of reproductive age

In a bivariate logistic regression analysis between underweight and WASH variables, latrine hygiene and use were significantly associated with underweight. Multivariate analysis was carried out between underweight and WASH variables separately after adjusting for sociodemographic characteristics of the women of reproductive age (age, occupation, educational status, sex of the head, and wealth index of the household). Latrine hygiene and use were significantly associated with underweight. The odds of being underweight among those who use clean latrine were 0.43 (95% CI: 0.20, 0.92) compared to those who use unclean latrine or do not have toilet facilities (Table 3).

**TABLE 3:** Association between underweight and WASH of women of reproductive age in Arba Minch HDSS (*n* = 422).

Variables	Underweight		COR	AOR (95% CI)
	No Frq. (%)	Yes Frq. (%)		
Water source				
Unimproved	94 (22.27)	10 (2.37)		
Improved	295 (69.91)	23 (5.45)	0.73	1.33 (0.51, 3.45)
Location of water source				
Outside premise	363 (86.02)	32 (7.58)		
Inside premise	26 (6.16)	1 (0.24)	0.44	0.49 (0.06, 3.92)
Water availability during visit time				
Not available	67 (15.88)	8 (1.9)		
Available	322 (76.3)	25 (5.92)	0.65	0.87 (0.34, 2.22)
Latrine hygiene and use				
Not clean or no latrine	129 (30.57)	19 (4.5)		
Clean and underuse	260 (61.61)	14 (3.32)	0.37	0.43 (0.2, 0.92)*
Quality of latrine construction				
Poor	227 (53.79)	20 (4.74)		
Good	162 (38.39)	13 (3.08)	0.91	1.23 (0.56, 2.69)
Handwashing facility				
Available	63 (14.93)	9 (2.13)		
Not available	326 (77.25)	24 (5.69)	0.52	0.47 (0.18, 1.19)
Common use of latrine by family members				
Yes	310 (73.46)	29 (6.87)		
No	79 (18.72)	4 (0.95)	0.54	0.48 (0.16, 1.44)
Usual use of soap/ashes while handwashing				
Yes	252 (59.72)	24 (5.69)		
No	137 (32.46)	9 (2.13)	0.69	0.44 (0.18, 1.08)

* *p*‐value < .05; AOR were adjusted for sociodemographic characteristics of the women (age, occupation, educational status, sex of the head, and wealth index of the household).

## 4 DISCUSSION

The objective of this study was to assess the association between WASH and the nutritional status of women of reproductive age. We categorized the WASH indicators into water source, location of the water source, water availability during visit time, latrine hygiene and use, quality of latrine construction, handwashing facility, common use of latrine by family members, and the usual use of soap/ashes while handwashing. Pipe water was the most common source of water for three‐fourths of the households, and only 2.6% of the households did not have latrine facilities. The 2016 Ethiopian demographic and health survey indicated that around half of the households in Ethiopia use improved water sources, and more than one‐third of the households do not have latrines (Csace, 2016).

The current study identified that the odds of being underweight among women of reproductive age who use clean latrine have reduced by 57% compared to those who use unclean latrine or do not have toilet facilities. This is consistent with a previous study conducted in Nepal (Rana et al., 2022), which revealed that the odds of underweight were reduced by 30% among women of reproductive age who were from households with improved toilet facilities. In addition, the result of this study was in line with the study done in India (Radhika et al., 2018), which stated that unimproved WASH, particularly unimproved sanitation, was associated with nearly two times increased odds of underweight among women. Moreover, it was consistent with a study conducted in Northwest Ethiopia (Wubetie & Mekonen, 2023), which revealed that the odds of undernutrition were sixfold increased among women with improved WASH, particularly latrine utilization. Poor WASH practices (water facilities outside the premises and unimproved sanitation) had a statistically significant association with women's nutritional status, according to a study conducted in India (Chattopadhyay et al., 2019). Our finding is also in line with the study done in Nigeria (Morakinyo et al., 2020), which showed that the odds of being underweight are doubled among women of reproductive age living in households with unimproved household and environmental conditions.

Our finding did not show an association between the improvements of the water source, location of water source, water availability at home during a visit, quality of latrine construction, handwashing facility, common use of latrine by family members, and the use of soap/ashes while handwashing. Contrarily, an analysis based on the burden of underweight and overweight women in Addis Ababa, Ethiopia, among women based on three EDHS reports (2000, 2005, and 2011) showed that undernutrition was associated with those without access to an improved source of water and toilet facilities (Tebekaw et al., 2014). This may be due to the fact that the nutritional status of women of reproductive age may be significantly affected by an unimproved toilet facility that puts them at risk for infectious illness and leads to undernutrition when the prevalence of poor WASH is high (Morakinyo et al., 2020; Rana et al., 2022; Van Minh & Hung, 2011). Unhealthy household environments, including unclean drinking water and poor toilet facilities, can lead to various illnesses, affecting nutritional status by causing appetite loss and nutrient absorption (Wubetie & Mekonen, 2023). However, our findings did not prove this hypothesis except for latrine use and cleanliness.

### 4.1 Strengths and limitations

The study was focused specifically on the association between WASH and undernutrition among women of reproductive age group. It was a community‐based, cross‐sectional design with randomly selected women of reproductive age using the Arba Minch HDSS database. The HDSS was established to cover the three agroecologies (lowland, midland, and highland) of Ethiopia, which have a significant effect on the dietary practices of the population. We tried to exclude pregnant and recently delivered women by using a checklist, for whom the use of BMI to determine nutritional status is not feasible. The cross‐sectional design makes it difficult to determine the cause‐and‐effect relationship. We included separate aspects of WASH as a main explanatory variable rather than a single index, which will help the readers to consider a practical point of view on the components of WASH. For the analysis, we considered a separate WASH variable.

## 5 CONCLUSION

The utilization of clean latrine facilities is negatively associated with being underweight among women of reproductive age. In places where there are improvements in WAHS, including a higher proportion of household use of improved water sources and higher latrine coverage, the association of most of the components of WASH with the nutritional status of the adult population may not be observed. From WASH components, latrine utilization and keeping the latrine clean to use need to be focussed to reduce the risk of being underweight among women of reproductive age.

## AUTHOR CONTRIBUTIONS

**Tamirat Gezahegn Guyo:** Conceptualization (supporting); data curation (equal); formal analysis (equal); funding acquisition (supporting); investigation (equal); methodology (equal); project administration (supporting); resources (supporting); software (supporting); supervision (supporting); validation (supporting); visualization (equal); writing – original draft (equal); writing – review and editing (equal). **Melesse Mengesha Merkina:** Conceptualization (equal); data curation (equal); formal analysis (equal); funding acquisition (lead); investigation (equal); methodology (equal); project administration (lead); resources (equal); software (equal); supervision (equal); validation (equal); visualization (equal); writing – original draft (equal); writing – review and editing (equal). **Desta Haftu Hayelom:** Conceptualization (equal); data curation (supporting); formal analysis (supporting); funding acquisition (supporting); investigation (supporting); methodology (equal); project administration (supporting); resources (supporting); software (supporting); supervision (supporting); validation (supporting); visualization (supporting); writing – original draft (supporting); writing – review and editing (equal). **Darik Temesgen Assefa:** Conceptualization (supporting); data curation (equal); formal analysis (equal); funding acquisition (supporting); investigation (equal); methodology (equal); project administration (supporting); resources (equal); software (supporting); supervision (equal); validation (equal); visualization (supporting); writing – original draft (supporting); writing – review and editing (equal). **Befikadu Tariku Gutema:** Conceptualization (equal); data curation (equal); formal analysis (equal); funding acquisition (supporting); investigation (supporting); methodology (equal); project administration (supporting); resources (supporting); software (equal); supervision (supporting); validation (supporting); visualization (equal); writing – original draft (equal); writing – review and editing (equal).

## CONFLICT OF INTEREST STATEMENT

The authors declare no conflicts of interest.

## Data Availability

The datasets used and/or analyzed during the current study are available from the corresponding author on reasonable request.
